# Supplementary material for: Genetically encoded transcriptional plasticity underlies stress adaptation in Mycobacterium tuberculosis
Source: Nat Commun. 2024 Apr 10;15:3088. doi: 10.1038/s41467-024-47410-5 (PMC11006872; doi:10.1038/s41467-024-47410-5)
Supplement: Supplementary file 1 — Supplementary information [file 41467_2024_47410_MOESM1_ESM.pdf]

Supplementary Information for

Genetically encoded transcriptional plasticity underlies stress adaptation in Mycobacterium tuberculosis

Cheng Bei[1#], Junhao Zhu[2,3#], Peter H Culviner[2], Mingyu Gan[4], Eric J. Rubin[2], Sarah M Fortune[2], Qian Gao[1,5*], Qingyun Liu[2,6*]

[1]Key Laboratory of Medical Molecular Virology (MOE/NHC/CAMS), School of Basic Medical Science, Shanghai Medical College, Shanghai Institute of Infectious Disease and Biosecurity, Fudan University, Shanghai, China.

[2]Department of Immunology and Infectious Diseases, Harvard T. H. Chan School of Public Health, Boston, Massachusetts, USA.

[3]CAS Key Laboratory of Pathogen Microbiology and Immunology, Institute of Microbiology, Chinese Academy of Sciences, Beijing, China

[4]Center for Molecular Medicine, Children's Hospital of Fudan University, National Children's Medical Center, Shanghai, 201102, China

[5]National Clinical Research Center for Infectious Diseases, Shenzhen Third People's Hospital, Shenzhen, Guangdong Province, China

[6]Current affiliation: Department of Genetics, University of North Carolina at Chapel Hill, Chapel Hill, NC 27599, USA.

[#]These authors contributed equally: Cheng Bei, Junhao Zhu.

[*]Correspondence: Qian Gao (qiangao@fudan.edu.cn), Qingyun Liu (qingyun_liu@med.unc.edu)

This PDF file includes:

Figure S1 to S9

Figure S1.

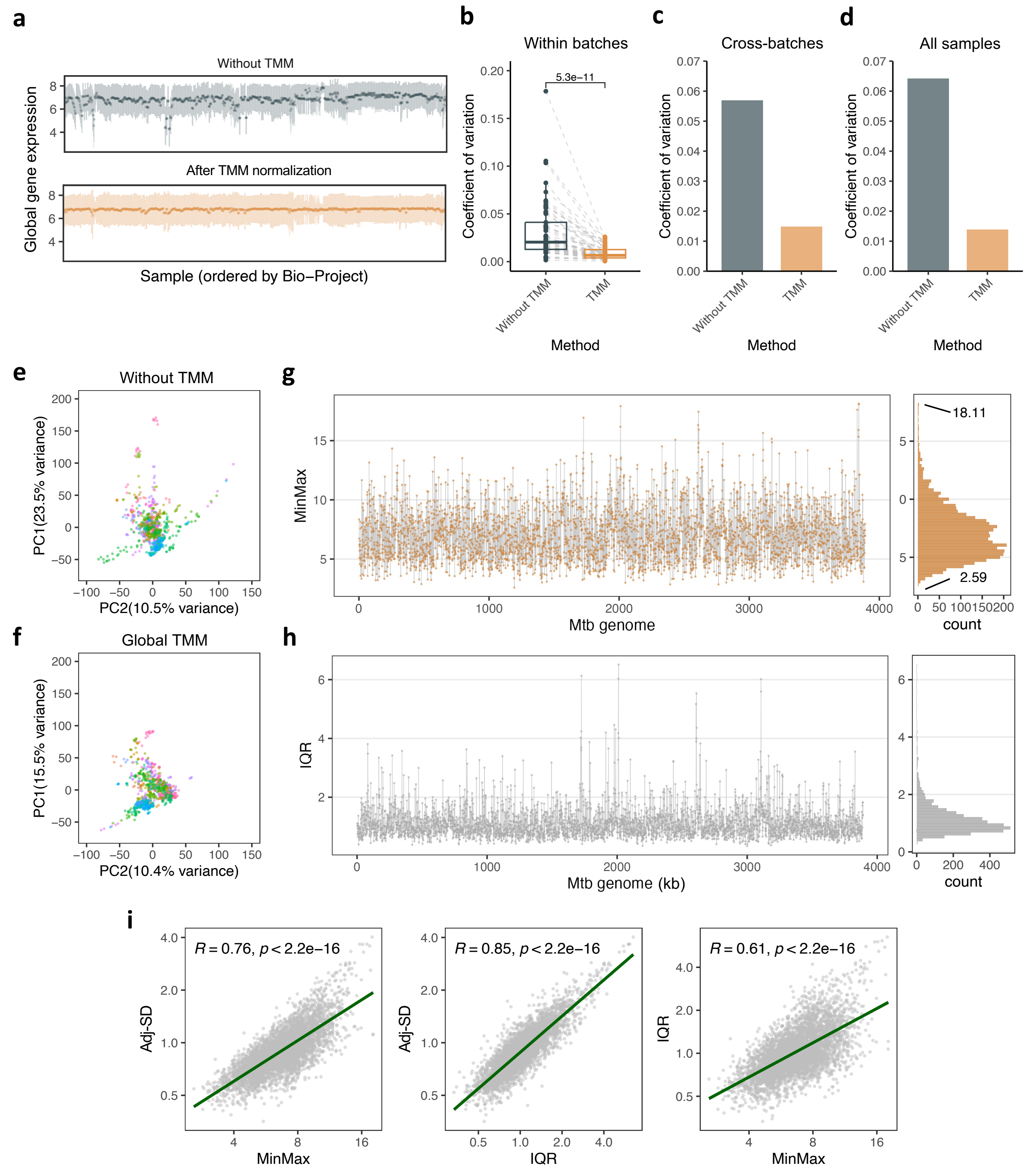

Supplementary Figure 1 (a) Global gene expression distribution of 894 samples before and after TMM normalization. Global gene expression distribution for each sample is presented by median ± IQR of expression level of global genes. **(b)** Coefficient of variation (CV) of global genes' median expression level across different samples in each batch (i.e., Bio-project). Paired Wilcoxon test is presented. **(c)** CV of global genes' median expression level across different batches. **(d)** CV of global genes' median expression level across all 894 samples. **(e-f)** PCA of transcriptional profiles of all 894 samples before (e) and after (f) TMM normalization. Samples belong to the same Bio-project are subjected to the same color. **(g)** Genome-wide *MinMax* profiles of the 3,891 *Mtb* gene, ordered by their genomic position. The genome-wide distributions of *MinMax* are illustrated in the right panel, with the highest and lowest *MinMax* values being 18.11 and 2.59 respectively. **(h)** Genome-wide *IQR* profiles of the 3,891 *Mtb* genes, ordered by their genomic position. The genome-wide distributions of *IQR* are illustrated in the right panel. **(i)** Correlations among adj-*SD*, *MinMax* and *IQR*, with green lines denoting linear fits.

Figure S2.

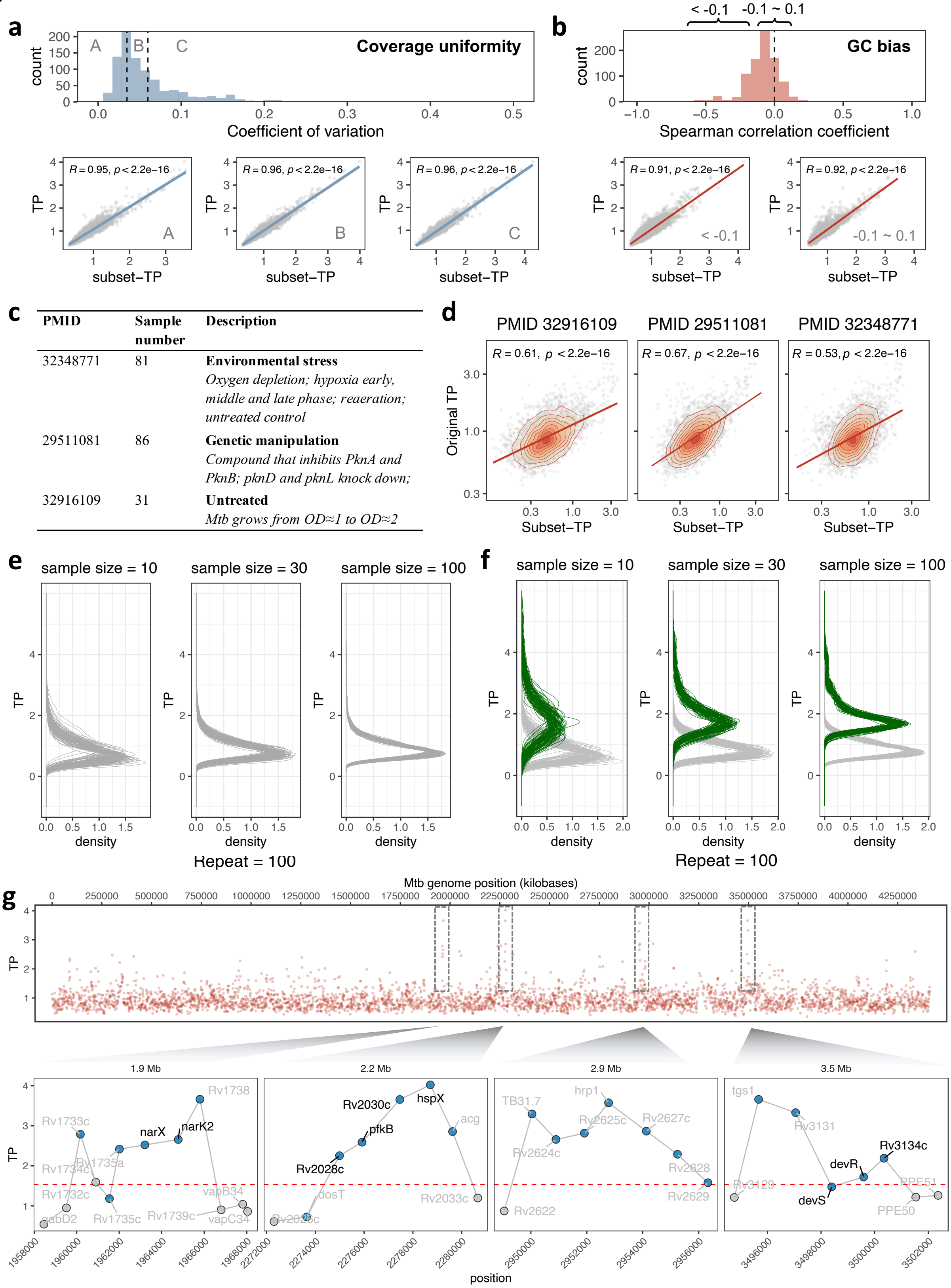

PMID	Sample number	Description
32348771	81	**Environmental stress** *Oxygen depletion; hypoxia early, middle and late phase; reaeration; untreated control*
29511081	86	**Genetic manipulation** *Compound that inhibits PknA and PknB; pknD and pknL knock down;*
32916109	31	**Untreated** *Mtb grows from OD≈1 to OD≈2*

Supplementary Figure 2 (a) Distribution of read coverage uniformity of 894 samples (top). Gene body coverage profiles were calculated as specified in *Materials and Methods*. For each sample, we took its gene body coverage profile across the 100 interpolated positions and calculated its Coefficient of Variation. TP calculated by sub-samples with similar degrees of coverage uniformity shows significant correlations with the original TP calculated by the total 894 samples (bottom). **(b)** Distribution of GC bias of 894 samples (top). TP calculated by sub-samples with high degree of GC bias ($R < -0.1$) or low degree of GC bias ($-0.1 \le R < 0.1$) shows significant correlations with the original TP calculated by the total 894 samples (bottom). **(c)** The samples from three independent studies were selected to measure batch-specific TP. **(d)** The TP calculated with the samples from each individual study (subset-TP) is significantly correlated with original TP based on the whole dataset. **(e)** Density plots demonstrate the distributions of TP estimated using 10, 30 and 100 randomly selected samples. This bootstrap analysis was performed 100 times for each sampling size, and all repeats are overlaid in each plot. **(f)** Density plots show the TP distributions of 195 high-TP genes (green) in Fig 2a and the remaining 3,696 genes (grey) at each sample size in the resampling process in Fig. S2d. This bootstrap analysis was repeated 100 times for each sampling size, with all repeats overlapped in each plot. **(g)** A zoom-in view of the four high-TP hotspots in Fig. 1f (top). Black texts represent operonic genes, and blue dots represent genes in DosR regulon (bottom).

Figure S3.

a

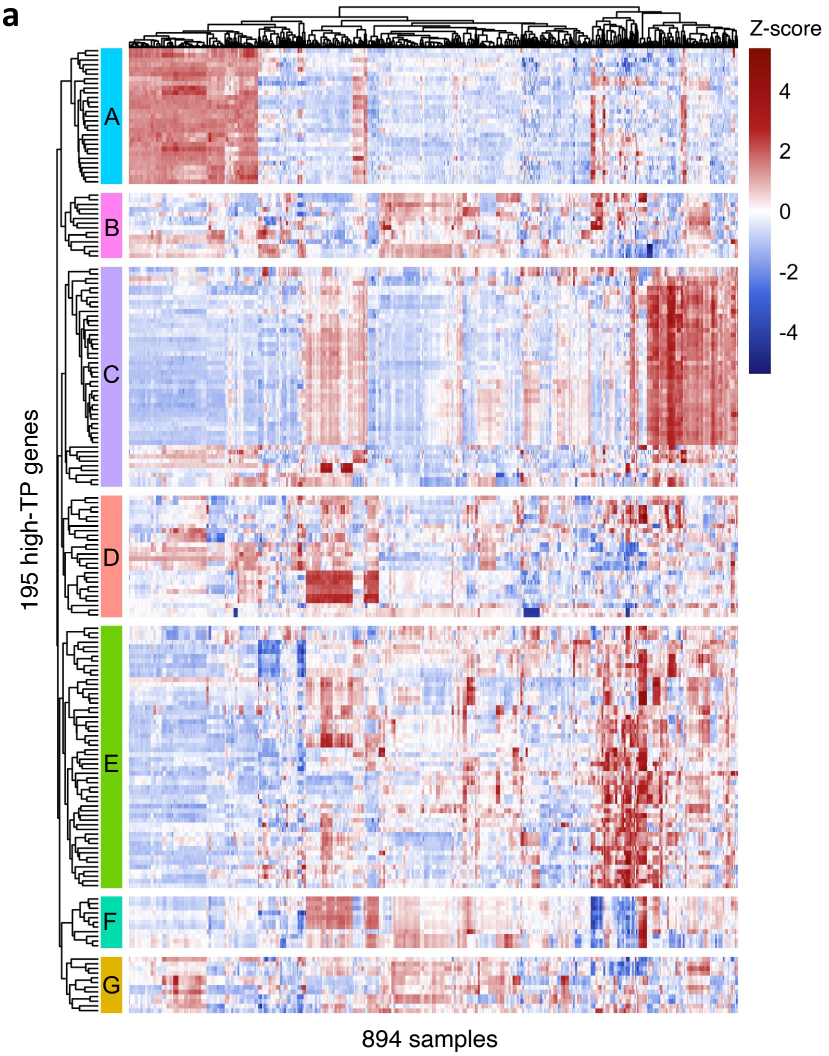

b

Cluster	Term	Gene number	FDR
A	Nuclease	3	0.016
	Lipoprotein	4	0.021
	Maintenance of CRISPR repeat elements	2	0.048
B	Cell redox homeostasis	3	0.034
C	Response to hypoxia	12	$7.04*10^{-11}$
	Universal stress protein A	6	$7.23*10^{-7}$
	Rossmann-like alpha/beta/alpha sandwich fold	6	$4.75*10^{-4}$
	Response to host immune response	5	0.036
	Nitroreductase-like	3	0.049
D	NA		
E	Response to copper ion	5	$3.62*10^{-4}$
	Biosynthesis of siderophore group nonribosomal peptides	4	$4.90*10^{-4}$
	Winged helix-turn-helix DNA-binding domain	5	0.005
	Cytoplasm	15	0.005
	ArsR-type helix-turn-helix	4	0.014
	Protein of unknown function DUF1490	3	0.018
	Response to cadmium ion	3	0.021
F	Phenolic phthiocerol biosynthetic process	3	$1.46*10^{-4}$
G	Alkane catabolic process	2	0.022

Supplementary Figure 3 (a) A Heatmap displaying the expression level of 195 high-TP genes across the 894 samples. 195 genes are grouped to 7 clusters based on k-means method. Expression levels (log RPKM) are scaled and normalized using the Z-score method. **(b)** Enrichment analysis of genes in 7 cluster corresponding to Fig. S3a.

Figure S4.

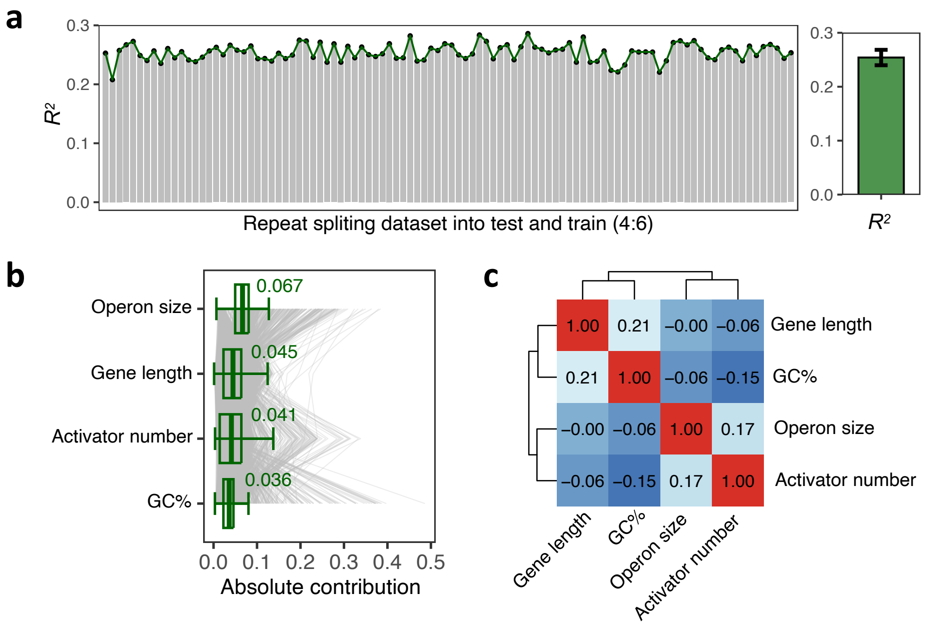

Supplementary Figure 4 (a) A barchart depicts the coefficients of determination (R^2) of the 100 prediction models described in Fig. 3b. Each model was trained on data from 60% of randomly selected genes and the R^2 was calculated using the remaining data. The average of the 100 R^2 measures are shown in the right panel. The error bar represents the mean ± SD. **(b)** Boxplots illustrate the contribution of the four features to the SVM model described in Fig. 3d. The contribution of each feature to the SVM model is evaluated using the Shapley additive explanations (SHAP) method, and the absolute SHAP contribution scores were plotted (see *Materials and Methods*). Grey lines represent the 2,016 genes involved in the training of the SVM model in Fig. 3d. Green texts represent the median value of absolute contribution for each feature. **(c)** A heatmap shows the pairwise Spearman's correlation coefficients among the four impactful genetic features.

Figure S5.

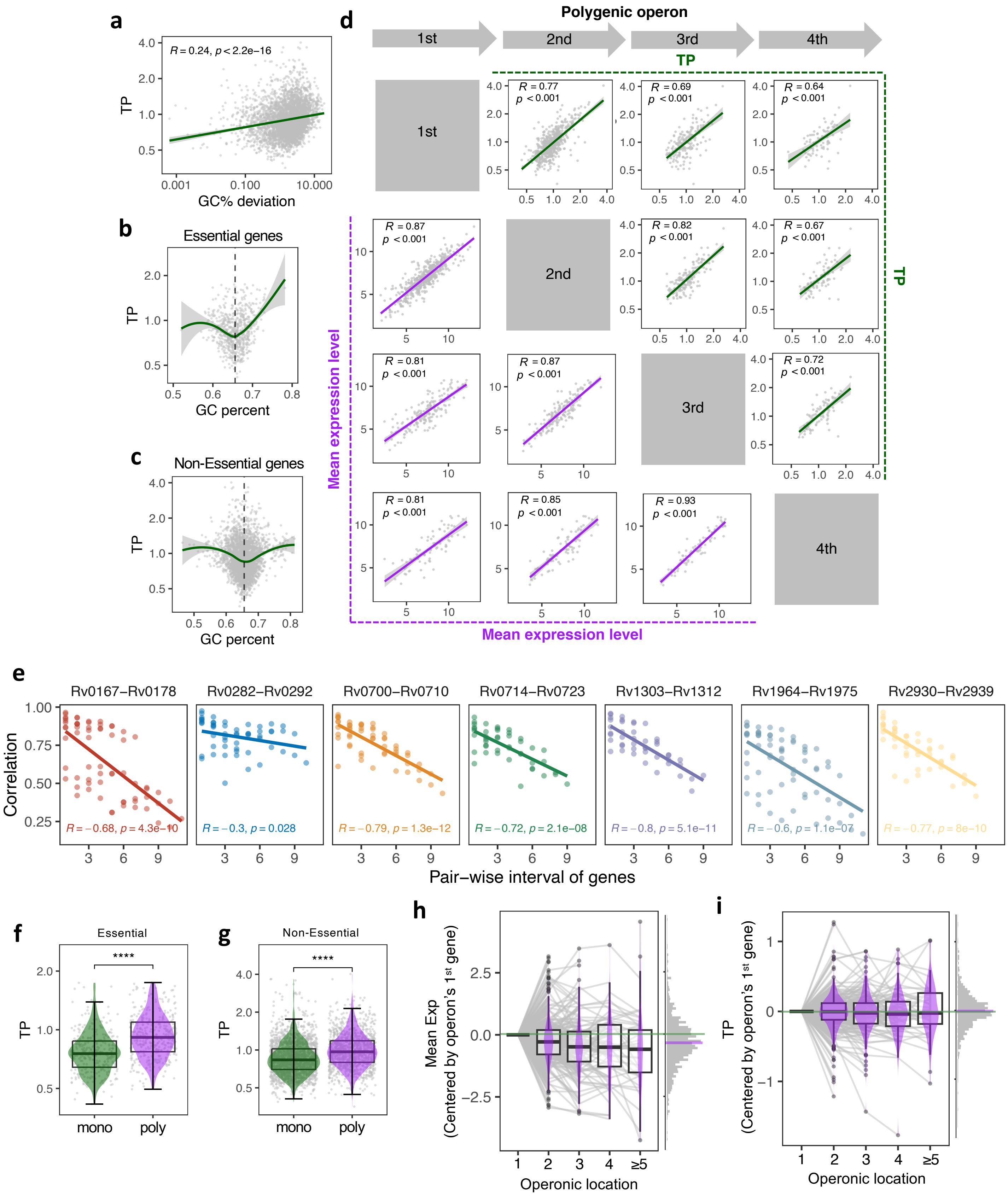

a
R = 0.24, p < 2.2e−16
TP
GC% deviation
b
Essential genes
TP
GC percent
c
Non-Essential genes
TP
GC percent
d
Polygenic operon
1st
2nd
3rd
4th
TP
R = 0.77 p < 0.001
R = 0.69 p < 0.001
R = 0.64 p < 0.001
R = 0.87 p < 0.001
R = 0.82 p < 0.001
R = 0.67 p < 0.001
R = 0.81 p < 0.001
R = 0.87 p < 0.001
R = 0.72 p < 0.001
R = 0.81 p < 0.001
R = 0.85 p < 0.001
R = 0.93 p < 0.001
Mean expression level
Mean expression level
e
Rv0167−Rv0178
Rv0282−Rv0292
Rv0700−Rv0710
Rv0714−Rv0723
Rv1303−Rv1312
Rv1964−Rv1975
Rv2930−Rv2939
Correlation
R = −0.68, p = 4.3e−10
R = −0.3, p = 0.028
R = −0.79, p = 1.3e−12
R = −0.72, p = 2.1e−08
R = −0.8, p = 5.1e−11
R = −0.6, p = 1.1e−07
R = −0.77, p = 8e−10
Pair−wise interval of genes
f
Essential
****
TP
mono
poly
g
Non-Essential
****
TP
mono
poly
h
Mean Exp (Centered by operon's 1st gene)
Operonic location
i
TP (Centered by operon's 1st gene)
Operonic location

Supplementary Figure 5 (a) GC% deviation exhibits a significant positive correlation with TP. The GC% deviation is defined as the absolute difference between a gene's GC% and the genome-wide GC%. **(b-c)** Scatter plots illustrate the correlations between TP and GC% in essential genes (b) and non-essential genes (c). Green lines represent the LOESS fits, while black dashed lines represent the genome-wide GC content (65.6%). **(d)** Pair-wise comparisons of TPs and mean expression values among genes located in different positions (1st, 2nd, 3rd, and 4th from the 5' termini of the transcripts, respectively) of polygenic operons. Colored lines represent the linear fits (purple for mean expression level and green for TP). **(e)** Two genes that are closer to each other have more similar expression level. X-axis represents the distance between two operonic genes, e.g., the interval of adjacent genes is 1. Y-axis represents the Pearson's correlation coefficient of gene expression level between two genes. Pearson's correlation coefficient and corresponding p value are presented. **(f-g)** Boxplots demonstrate that both essential (f) and non-essential (g) genes in polygenic operons have significantly higher TP than genes in monogenic operons. Shaded areas depict the TP distribution of monogenic operon genes (green) and polygenic operon genes (purple). Error bars represent median $\pm$ 1.5 $\times$ IQR. The X-axis in the bottom plot is log-scaled. **** p value < 0.0001. **(h-i)** Boxplots show centered mean expression values (h) and TP (i) of each gene in its operon. The X-axis indicates the location of each gene in its operon. Mean expression values or TPs of co-operonic genes are centered by the mean expression or TP of the first gene in this operon. The normalized mean expression levels or TPs of adjacent operonic genes are connected by grey lines to demonstrate the trend of expression or TP changes along this operon. Density plots represent the aggregated distribution of normalized expression or TP values. Purple lines represent the median values of all genes, and green solid lines represent that of the leading genes (centered to become zero).

Figure S6.

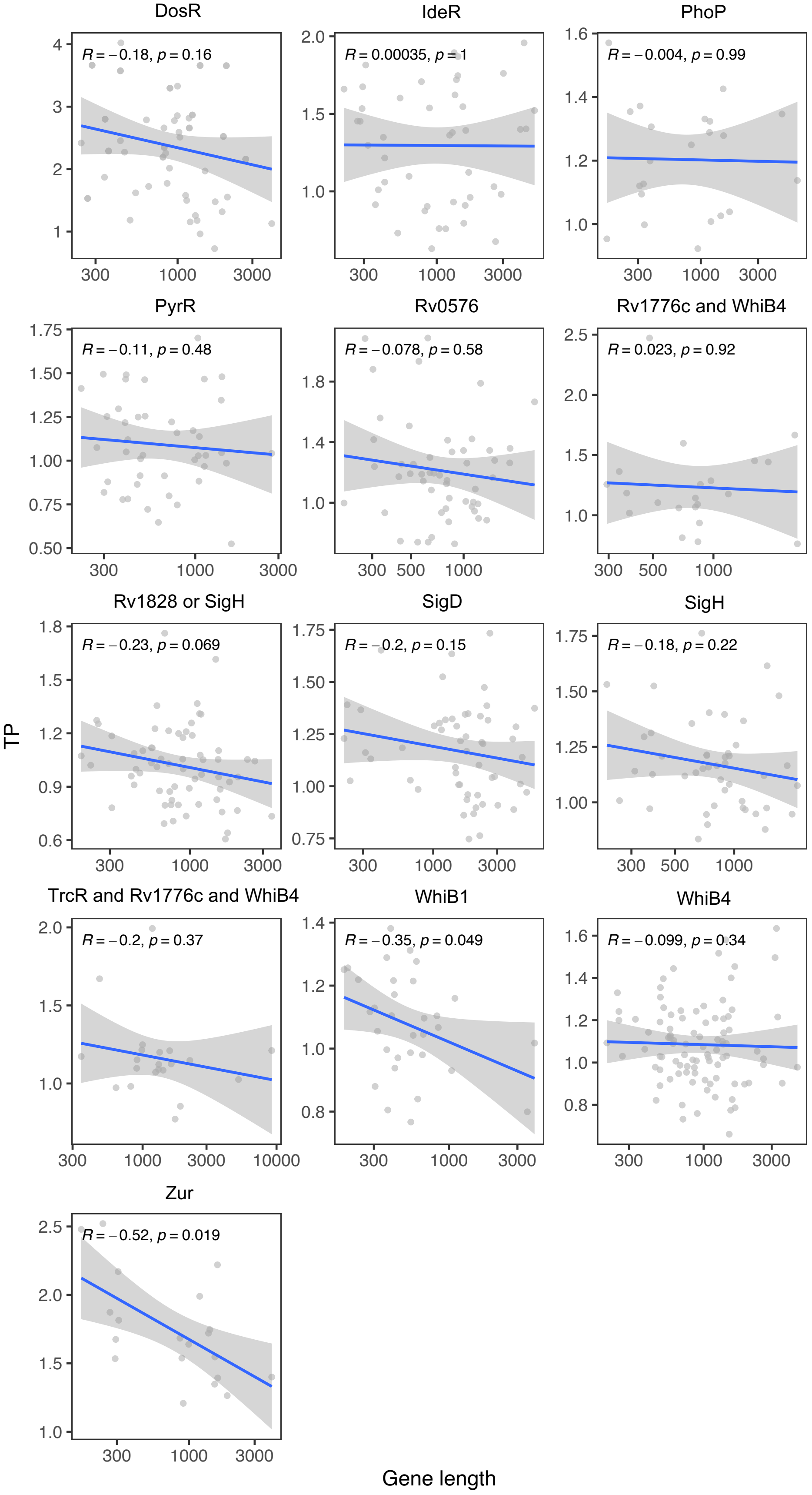

Supplementary Figure 6 Scatter plots illustrate the correlations between TP and gene width in 13 regulons, each containing more than 20 genes (“DosR-1” and “DosR-2” were arbitrarily consolidated into the DosR regulon). Lines represent linear fits. Spearman’s correlation coefficient and corresponding *p* values are provided for each regulon.

Figure S7.

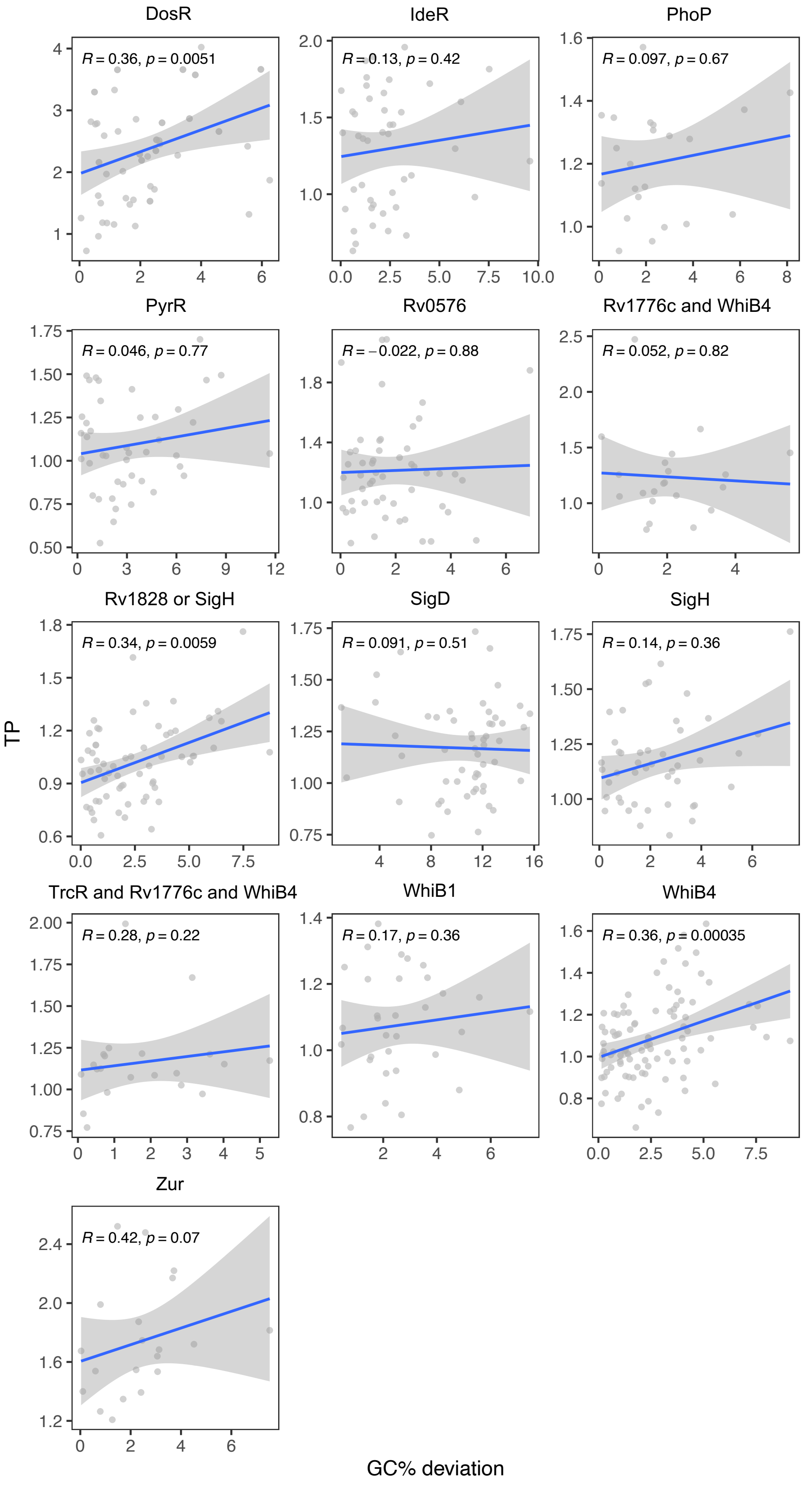

Supplementary Figure 7 Scatter plots illustrate the correlations between TP and GC% deviation in the 13 regulons described in Fig. S6. Lines represent linear fits. Spearman's correlation coefficient and corresponding *p* values are provided for each regulon.

Figure S8.

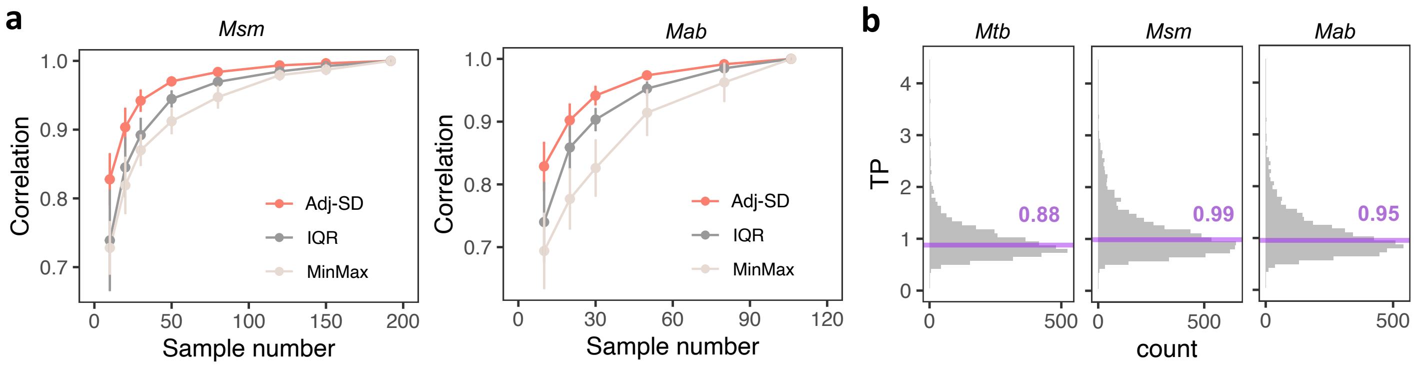

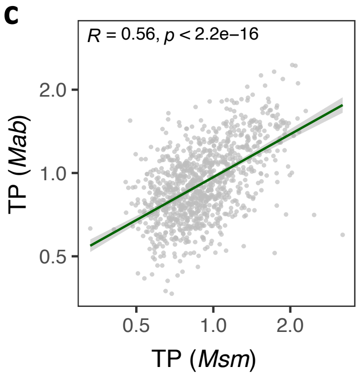

d

Term (Msm)	FDR	Count
Universal stress protein A	0.0014	7
Iron	0.0027	22
Oxidoreductase	0.0034	50
Metal ion binding	0.0106	26
Stress response	0.0303	5
Membrane	0.0380	58
Peroxidase	0.0993	6

e

Term (Mab)	FDR	Count
Ferritin-like superfamily	0.0033	9
Oxidoreductase	0.0041	36
Membrane	0.0262	44

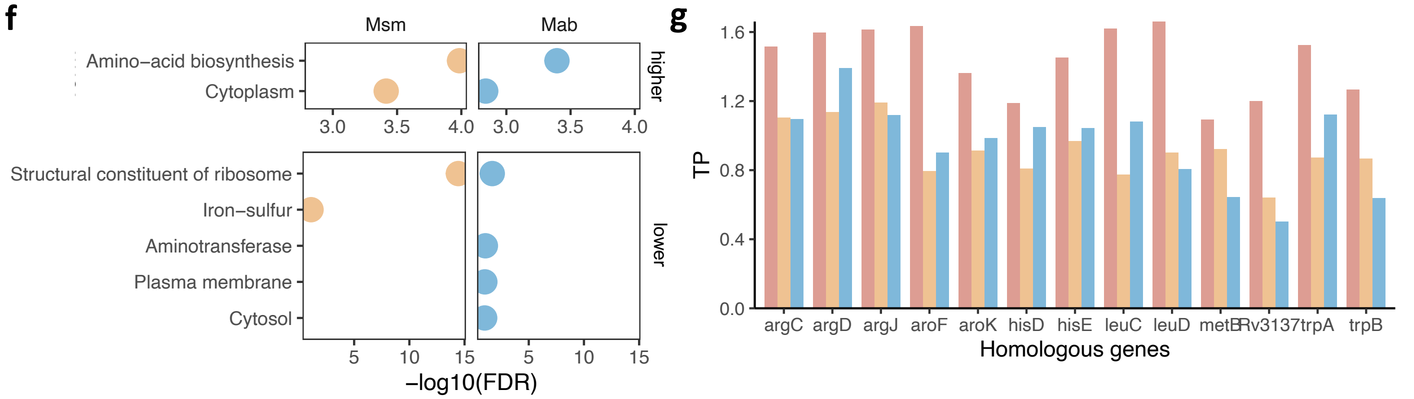

Supplementary Figure 8 (a) Comparisons of *adj-SD*, *IQR*, and *MinMax* metrics in describing expression variability of *Msm* and *Mab* genes were made using a bootstrap analysis analogous to the approach described in Fig. 1e (see *Materials and Methods*). The lines and the error bars denote the means and the standard deviations of the correlation coefficients rendered by the bootstrap analysis using different metrics. **(b)** TP distribution profiles of *Mtb*, *Msm*, and *Mab* genes. Purple lines represent the median TP of all genes for each species. **(c)** Significant correlations of TPs between homologous genes in *Msm* and *Mab* (see *Materials and Methods*). **(d-e)** Enrichment analysis conducted on the top 5% highest TP genes in *Msm* (d) and *Mab* (e) and using the DAVID platform. The significance threshold for enrichment results was set at FDR < 0.1. **(f)** Enrichment analysis of top 5% *Mtb* homologous genes that have higher (top) or lower (bottom) TP than *Msm* and *Mab*. **(g)** TP of amino-acid synthesis genes is higher in *Mtb* (red) than *Msm* (orange) and *Mab* (blue).

Figure S9.

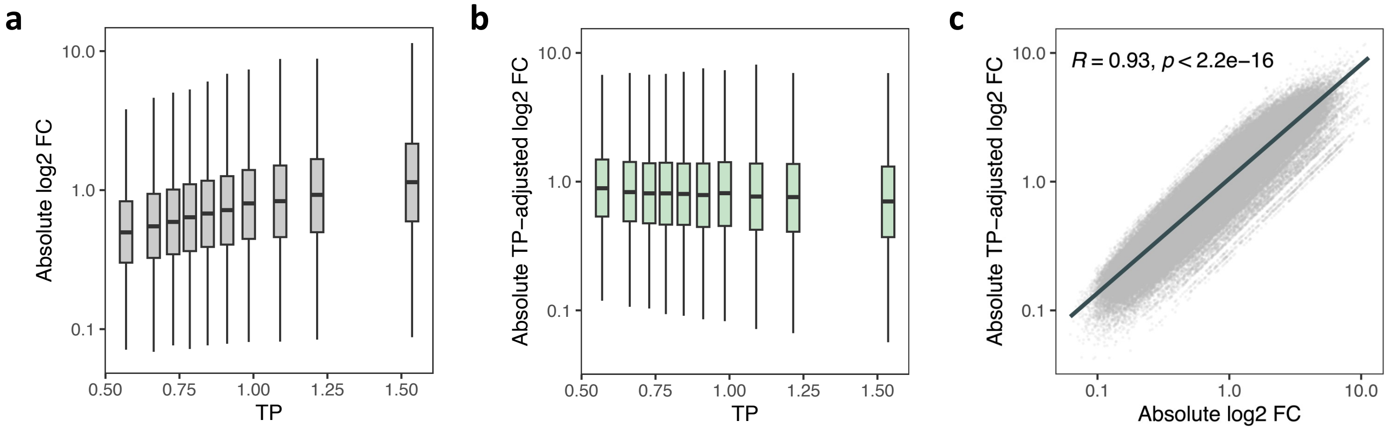

Supplementary Figure 9 (a-b) Correlation between TP and absolute log2 FC values (a) and absolute TP-adjusted log2 FC values (b) in 127 differential expression analyses (see *Materials and Methods*). Genes are binned to 10 bins with equal size according to their TPs. Box plots represent median ± 1.5 × IQR. **(c)** Correlation between absolute log2 FC and TP-adjusted log2 FC.
