# Supplementary material for: Genetically encoded transcriptional plasticity underlies stress adaptation in Mycobacterium tuberculosis
Source: Nat Commun. 2024 Apr 10;15:3088. doi: 10.1038/s41467-024-47410-5 (PMC11006872; doi:10.1038/s41467-024-47410-5)
Supplement: Supplementary file 3 — Description of Additional Supplementary Files [file 41467_2024_47410_MOESM3_ESM.pdf]

Description of Additional Supplementary Files

Supplementary Data 1 : RNA-seq samples of *M. tuberculosis, M. smegmatis,* and *M. abscessus* included in this study.

Supplementary Data 2 : Expression profile (TMM normalized RPKM) of 3,891 *M. tuberculosis* genes in 894 samples.

Supplementary Data 3 : Descriptive statistics of gene expression distribution for each *M. tuberculosis* gene in 894 samples.

Supplementary Data 4 : Functional enrichment results of 195 high-TP *M. tuberculosis* genes.

Supplementary Data 5 : A list of 119 features of *M. tuberculosis* genes.

Supplementary Data 6 : A list of 36 gene regulons of *M. tuberculosis*.

Supplementary Data 7 : TP of *M. smegmatis* and *M. abscessus* genes.

Supplementary Data 8 : 95th and 5th expression levels for each *M. tuberculosis* gene in 894 samples and their log2 fold changes compared to gene's mean expression level.
