# Supplementary material for: Genetically encoded transcriptional plasticity underlies stress adaptation in Mycobacterium tuberculosis
Source: Nat Commun. 2024 Apr 10;15:3088. doi: 10.1038/s41467-024-47410-5 (PMC11006872; doi:10.1038/s41467-024-47410-5)
Supplement: Supplementary file 12 — Reporting Summary [file 41467_2024_47410_MOESM12_ESM.pdf]

nature portfolio

Corresponding author(s): Qingyun Liu
Qian Gao

Last updated by author(s): Feb 14, 2024

Reporting Summary

Nature Portfolio wishes to improve the reproducibility of the work that we publish. This form provides structure for consistency and transparency in reporting. For further information on Nature Portfolio policies, see our Editorial Policies and the Editorial Policy Checklist.

Statistics

For all statistical analyses, confirm that the following items are present in the figure legend, table legend, main text, or Methods section.

n/a	Confirmed	
[ ]	[x]	The exact sample size (*n*) for each experimental group/condition, given as a discrete number and unit of measurement
[ ]	[x]	A statement on whether measurements were taken from distinct samples or whether the same sample was measured repeatedly
[ ]	[x]	The statistical test(s) used AND whether they are one- or two-sided<br>*Only common tests should be described solely by name; describe more complex techniques in the Methods section.*
[x]	[ ]	A description of all covariates tested
[ ]	[x]	A description of any assumptions or corrections, such as tests of normality and adjustment for multiple comparisons
[ ]	[x]	A full description of the statistical parameters including central tendency (e.g. means) or other basic estimates (e.g. regression coefficient) AND variation (e.g. standard deviation) or associated estimates of uncertainty (e.g. confidence intervals)
[ ]	[x]	For null hypothesis testing, the test statistic (e.g. *F*, *t*, *r*) with confidence intervals, effect sizes, degrees of freedom and *P* value noted<br>*Give P values as exact values whenever suitable.*
[x]	[ ]	For Bayesian analysis, information on the choice of priors and Markov chain Monte Carlo settings
[x]	[ ]	For hierarchical and complex designs, identification of the appropriate level for tests and full reporting of outcomes
[ ]	[x]	Estimates of effect sizes (e.g. Cohen's *d*, Pearson's *r*), indicating how they were calculated

Our web collection on statistics for biologists contains articles on many of the points above.

Software and code

Policy information about availability of computer code

Data collection	RNA-seq data were obtained from NCBI SRA database. Gene features were retrieved from published articles.
Data analysis	FASTQ files of all 1,084 samples were downloaded using Fastq-dump (version 2.8.0). Adaptor trimming and the removal of low-quality sequencing reads were conducted using Trimmomatic (version 0.39). The filtered profiles were then mapped against the H37Rv reference genome (ASM19595v2) using Bowtie2 (version 2.2.9), and duplicated reads were removed with SAMtools (version 1.9). TP analyses were done in R (version 4.0.2) and Python (3.11). LGBM model was achieved using the Python-compiled lightgbm package (version 3.3.2).

For manuscripts utilizing custom algorithms or software that are central to the research but not yet described in published literature, software must be made available to editors and reviewers. We strongly encourage code deposition in a community repository (e.g. GitHub). See the Nature Portfolio guidelines for submitting code & software for further information.

Data

Policy information about availability of data

All manuscripts must include a data availability statement. This statement should provide the following information, where applicable:

- Accession codes, unique identifiers, or web links for publicly available datasets
- A description of any restrictions on data availability
- For clinical datasets or third party data, please ensure that the statement adheres to our policy

No primary data has been generated in this study. RNA-Seq data sources are listed in Supplementary Data 1. The conditions of 894 samples are annotated in Supplementary Data 1. The integrated transcriptional profile containing 3,891 genes and 894 samples is available in Supplementary Data 2. TP and descriptive

statistics for gene expression levels are available in Supplementary Data 3. Collected genetic features are listed in Supplementary Data 4. High-TP genes and their enrichment results are listed in Supplementary Data 5. Regulon genes of Mtb are listed in Supplementary Data 6. TP data of Msm and Mab are available in Supplementary Data 7. Benchmark of DEGs based on TP data of Mtb are shown in Supplementary Data 8. Source data are provided with this paper (https://github.com/ChengBEI-FDU/Transcriptional_Plasticity/tree/main/source_data).

Research involving human participants, their data, or biological material

Policy information about studies with human participants or human data. See also policy information about sex, gender (identity/presentation), and sexual orientation and race, ethnicity and racism.

Reporting on sex and gender	NA
Reporting on race, ethnicity, or other socially relevant groupings	NA
Population characteristics	NA
Recruitment	NA
Ethics oversight	NA

Note that full information on the approval of the study protocol must also be provided in the manuscript.

Field-specific reporting

Please select the one below that is the best fit for your research. If you are not sure, read the appropriate sections before making your selection.

[x] Life sciences [ ] Behavioural & social sciences [ ] Ecological, evolutionary & environmental sciences

For a reference copy of the document with all sections, see nature.com/documents/nr-reporting-summary-flat.pdf

Life sciences study design

All studies must disclose on these points even when the disclosure is negative.

Sample size	We did not generate new sequencing data in this study. All the data used for analysis were obtained from previous studies. We used the keyword “tuberculosis” to search for publicly available RNA-Seq data of Mtb released on NCBI Sequence Read Archive (SRA) before January 1, 2022, and obtained a total of 1,084 datasets from 64 BioProjects with 47 associated research articles. After filtering criteria, a total of 894 samples were included in this study. The details of filtering criteria were described in the Materials and Methods.
Data exclusions	Samples with small library size (< 1,000,000 reads) and from Mtb strains other than H37Rv were excluded. Small genes (<=150bp), non-coding transcripts (tRNA, rRNA, and annotated non-coding RNAs in the Mtb genome) as well as non-expressing genes (read counts in all samples were zero) were removed. Samples from all three mycobacteria with a high proportion of zero-expressing genes (> 4% of total genes), and genes with low SI (SI < 6.5 in Mtb, < 4 in Msm and Mab) and genes that are not expressed in more than 1% of total samples are also excluded.
Replication	All the analyses can be reproduced by using the codes with deposited on Github.
Randomization	For TP estimation in bootstrapping analysis, we randomly selected different numbers of samples (10, 20, 30, 50, 100, 200, 300, 500, 800). This process has been repeated for 30 times. We trained the regression model in Fig. 3b by randomly selecting subset of 60% (2,335/3,891) of the total Mtb genes, and then used the model to predict the TPs of the remaining 40% (1,556/3,891) of Mtb genes. We iterated this process 100 times. Random sampling processes were done in R or Python. No covariate analysis was done.
Blinding	Authors were blinded to the allocations of samples as it is done by random sampling processes.

Reporting for specific materials, systems and methods

We require information from authors about some types of materials, experimental systems and methods used in many studies. Here, indicate whether each material, system or method listed is relevant to your study. If you are not sure if a list item applies to your research, read the appropriate section before selecting a response.

Materials & experimental systems

n/a	Involved in the study	
☒	☐	Antibodies
☒	☐	Eukaryotic cell lines
☒	☐	Palaeontology and archaeology
☒	☐	Animals and other organisms
☒	☐	Clinical data
☒	☐	Dual use research of concern
☒	☐	Plants

Methods

n/a	Involved in the study	
☒	☐	ChIP-seq
☒	☐	Flow cytometry
☒	☐	MRI-based neuroimaging

Plants

Seed stocks	NA
Novel plant genotypes	NA
Authentication	NA
